# A case of proliferative diabetic retinopathy in which scintillating particles appeared in the intravitreal cavity after laser photocoagulation

**DOI:** 10.1186/s12886-017-0654-5

**Published:** 2017-12-19

**Authors:** Ryohsuke Kohmoto, Takatoshi Kobayashi, Takaki Sato, Daisaku Kimura, Masanori Fukumoto, Kensuke Tajiri, Teruyo Kida, Tsunehiko Ikeda

**Affiliations:** 1 Department of Ophthalmology, Osaka Medical College, 2-7 Daigaku-machi, Takatsuki City, Osaka, 569-8686 Japan; 2 Department of Ophthalmology, Takatsuki Red Cross Hospital, Takatsuki-City, Osaka, Japan

**Keywords:** Proliferative diabetic retinopathy, Laser photocoagulation, Scintillating particl, Posterior vitreous gel, Crystallin

## Abstract

**Background:**

To report a case of proliferative diabetic retinopathy (PDR) exhibiting the appearance of scintillating particles presumed to be crystallin inside the intravitreal cavity after laser photocoagulation.

**Case presentation:**

A 56-year-old male patient presented at our outpatient clinic after becoming aware of decreased vision in his right eye. Ocular examination performed at the patient’s initial visit revealed a massive preretinal macular hemorrhage due to PDR in his right eye. Fundus fluorescein angiography revealed extensive retinal non-perfusion areas and neovascularization in both eyes. However, no opacity was observed in the intravitreal cavity of his left eye. Vitreous surgery was performed on the patient’s right eye after ultrasonic phacoemulsification aspiration and intraocular lens implantation. Post surgery, the corrected VA in that eye improved from 0.1 to 1.0. In correlation with the treatment performed on the patient’s right eye, we began panretinal photocoagulation on his left eye. Examination performed prior to the patient’s third session of panretinal photocoagulation revealed a large number of scintillating particles in the posterior vitreous gel in front of the retina. Examination via slit-lamp microscopy revealed that the particles were of varied hues, and closely resembled a ‘Christmas tree’ cataract. No posterior vitreous detachment was observed, and since these particles were situated as if captured in the posterior vitreous gel, no eye-movement-associated mobility of the particles was observed. Since the cloudiness was not severe enough to interfere with photocoagulation, additional photocoagulation was performed, and the patient is currently under observation. Six months have now passed since the fourth photocoagulation procedure was performed, and there has been no change in the state of the particles. Optical coherence tomography imaging revealed no change before and after the panretinal photocoagulation. The corrected VA in his left eye has remained at 1.0 during the postoperative follow-up period.

**Conclusions:**

We speculate that the production of crystallin in the retina in this case was triggered by the photocoagulation procedure performed for diabetic retinopathy.

## Background

Crystallin was initially reported as a constituent protein of the lens, with later reports indicating its expression not only in the lens but also in the ciliary body, the neural retina, and retinal pigment epithelium [1, 2]. It has previously been reported that the retinas of diabetes patients have higher-than-normal expressions of αA-crystallin, and that its production is induced by glycated proteins, i.e., the accumulation of advanced glycation end-products (AGE) in the eye [3]. Here, we report a case of proliferative diabetic retinopathy (PDR) in which a scintillating substance that appeared to be crystallin developed in the intravitreal cavity after laser photocoagulation.

## Case presentation

A 56-year-old male patient presented at our outpatient clinic after becoming aware of decreased visual acuity (VA) in his right eye. His past medical history and general physical health findings revealed that his father had diabetes and his mother had hypertension, and although diabetes had been indicated for several years previous, he had not undergone treatment and had elevated levels of HbA1c (over 10%).

**Fig. 1:**
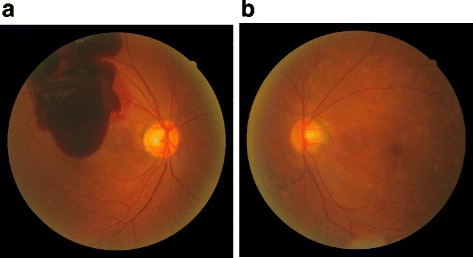
Fundus images obtained at the initial examination of the patient. A massive preretinal macular hemorrhage was observed in the patient’s right eye (**a**), yet no opacity was observed in the intravitreal cavity of the patient’s left eye (**b**)

**Fig. 2:**
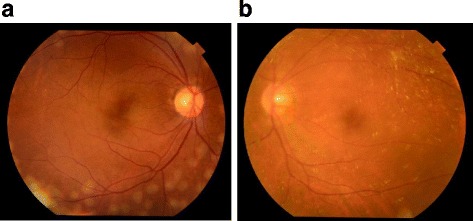
Fundus images obtained at the third photocoagulation procedure. The premacular hemorrhage was removed in the right eye, and the corrected visual acuity improved from 0.1 to 1.0 (**a**). In the left eye, a large number of scintillating particles were observed in the posterior vitreous gel in front of the retina **b**

**Fig. 3:**
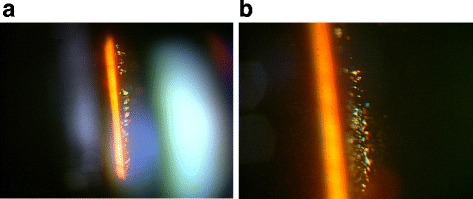
Slit-lamp microscopy image obtained at the third photocoagulation procedure. Numerous particles of various colors (i.e., yellow, red, and green) were observed (**a**). No posterior vitreous detachment had occurred, and the particles were captured in the posterior vitreous gel (**b**)

**Fig. 4:**
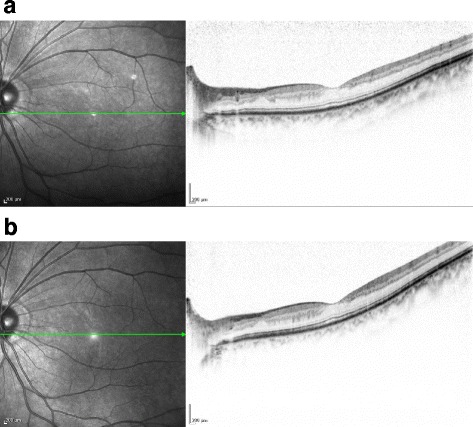
Optical coherence tomography (OCT) imaging obtained before and after the panretinal photocoagulation. The OCT imaging revealed no change before (**a**) and after (**b**) the panretinal photocoagulation

Ocular examination performed at the patient’s initial visit revealed a massive preretinal macular hemorrhage due to PDR in his right eye (Fig. 1a). Fundus fluorescein angiography revealed extensive retinal non-perfusion areas and neovascularization in both eyes. However, no opacity was observed in the intravitreal cavity of his left eye (Fig. 1b). Vitreous surgery was performed on the patient’s right eye after ultrasonic phacoemulsification aspiration and intraocular lens implantation. Post surgery, the corrected VA in that eye improved from 0.1 to 1.0 **(**Fig. 2a **)**. In correlation with the treatment performed on the patient’s right eye, we began panretinal photocoagulation on his left eye. Examination performed prior to the patient’s third session of panretinal photocoagulation revealed a large number of scintillating particles in the posterior vitreous gel in front of the retina (Fig. 2b). Examination via slit lamp biomicroscopy with contact lens revealed particles of various colors (i.e., yellow, red, and green) that in both shape and hue closely resembled the crystallin particles found in typical ‘Christmas tree’ type cataract (Fig. 3a, b). No posterior vitreous detachment was observed, and since these particles were situated as if captured in the posterior vitreous gel, no eye-movement-associated mobility of the particles was observed. Since the cloudiness was not severe enough to interfere with photocoagulation, additional photocoagulation was performed, and the patient is currently under observation. Six months have now passed since the fourth photocoagulation procedures was performed, and there has been no change in the state of the particles. Optical coherence tomography imaging revealed no change before and after the panretinal photocoagulation **(**Fig. 4a, b **)**. The corrected VA in his left eye remained 1.0 during follow-up period.

## Discussion

In vertebrates, crystallin proteins are classified into α, β, and γ subtypes, and α-crystallin reportedly plays a key role in various eye diseases [1, 2]. The α-crystallin protein consists of two different families of heat-shock proteins, i.e., αA and αB. α-crystallin was initially reported as a constituent protein of the lens of the eye, with later reports indicating its expression not only in the lens but also in the ciliary body, the neural retina, and retinal pigment epithelium [1, 2]. In addition, its expression is induced not only by thermal stimulation, but also by various other stress factors such as ischemia, hypoxia, and inflammation. It is also involved in cell differentiation, apoptosis, and controlling cell proliferation. Several previous reports have indicated that crystallin is detected in the vitreous body of various animal eyes [4–6], and although believed to be released primarily as the result of lens-capsule damage [7], it appears that it is possibly produced in other cells, such as retinal astrocytes [8].

It should be noted that numerous previous reports have indicated the involvement of crystallin in diabetic retinopathy (DR) [3, 9–13]. Kase et al. reported higher-than-normal expression of αA-crystallin in the retinas of diabetic-patient eyes even at the stage of no retinopathy, noting that its production is induced by the accumulation of glycated protein (i.e., AGE) in the eye [3]. It has also been reported that in the course of ocular neovascularization in mice, αB-crystallin binds to vascular endothelial growth factor (VEGF), thus suppressing the degradation of VEGF [10] Moreover, when the expression of α-crystallin is analyzed in the proliferative tissue obtained from DR cases, the expression of αB-crystallin reportedly can be observed in vascular endothelial cells of the retinal neovascularization, and its co-expression with VEGF has been confirmed by use of the double-staining method [11]. In this manner, crystallin seems to have increased expression in DR. Reports involving a diabetes animal model have also indicated that supplements such as taurine and vitamins A, B, and C work to inhibit the release of crystallin from the lens into the intravitreal cavity [12–15]. In addition to DR, crystallin is also reportedly involved in choroidal vasculature caused by age-related macular degeneration [16]. Moreover, other studies have reported elevated levels of intravitreal crystallin after retinal detachment, uveitis, and cataract surgery [17–20].

In this present case, while no scintillating particles were observed in the vitreous body at the beginning of the panretinal photocoagulation, they were observed at the time of the third photocoagulation procedure. Moreover, the particles were confined to the posterior vitreous gel in front of the retina, and were not found to be present in the anterior or central parts of the vitreous. These findings suggest that the particles were not released from the lens. Although synchysis scintillans is one of the differential diagnoses, it is a degenerative condition of the eye that results in liquefied vitreous humor and the accumulation of cholesterol crystals within the vitreous. Synchysis scintillans appears as small white floaters that freely move in the posterior part of the eye in the accompaniment of eye movement. The present case showed no liquefied vitreous humor and the particles were found to be stationary. Although no histological findings of the particles were obtained, we theorize that it is possible that inflammation of the retina as a result of photocoagulation may have increased the production of crystallin in the retina, whereupon it was released into the intravitreal cavity. The expression of various inflammatory cytokines in the retina can reportedly occur following laser photocoagulation [21], and the possibility that these cytokines enhance the production of crystallin in glial cells in the retina is certainly conceivable. In fact, Binz et al., who conducted a micro-array analysis after performing laser photocoagulation on the eyes of mice, reported an elevated expression of crystallin in the retina and retinal pigment epithelium [22]. In this present case, the underlying diabetes had not been previously treated, either medically or ophthalmologically, and this may have played a role in the rapid changes to the retinal environment caused by the laser photocoagulation. However, the reason why most DR eyes treated with PRP do not have scintillating particles remains unclear. In this present case, we performed panretinal photocoagulation for both eyes, however, only the left eye exhibited the appearance of scintillating particles. The reason for this is also unclear, yet we speculate that the presence of vitreous humor might have some effect on the appearance of scintillating particles.

## Conclusion

To the best of our knowledge, this is the first case in which scintillating particles were found to appear in the intravitreal cavity after laser photocoagulation for DR. Although these scintillating particles are symptomless and can possibly be followed-up without treatment, we believe that it is vital to investigate such changes in future cases.

## Abbreviations

AGE: Advanced glycation end-products
DR: Diabetic retinopathy
PDR: Proliferative diabetic retinopathy
VA: Visual acuity
VEGF: Vascular endothelial growth factor
